# Stochastic Games with Lexicographic Reachability-Safety Objectives

**DOI:** 10.1007/978-3-030-53291-8_21

**Published:** 2020-06-16

**Authors:** Krishnendu Chatterjee, Joost-Pieter Katoen, Maximilian Weininger, Tobias Winkler

**Affiliations:** 8 Microsoft Research Lab, Redmond, WA USA; 9 University of Southern California, Los Angeles, CA USA; 10 IST Austria, Klosterneuburg, Austria; 11 Technical University of Munich, Munich, Germany; 12 RWTH Aachen University, Aachen, Germany

## Abstract

We study turn-based stochastic zero-sum games with lexicographic preferences over reachability and safety objectives. Stochastic games are standard models in control, verification, and synthesis of stochastic reactive systems that exhibit both randomness as well as angelic and demonic non-determinism. Lexicographic order allows to consider multiple objectives with a strict preference order over the satisfaction of the objectives. To the best of our knowledge, stochastic games with lexicographic objectives have not been studied before. We establish determinacy of such games and present strategy and computational complexity results. For strategy complexity, we show that lexicographically optimal strategies exist that are deterministic and memory is only required to remember the already satisfied and violated objectives. For a constant number of objectives, we show that the relevant decision problem is in $\mathsf {NP}\cap \mathsf {coNP}$, matching the current known bound for single objectives; and in general the decision problem is $\mathsf {PSPACE}$-hard and can be solved in $\mathsf {NEXPTIME}\cap \mathsf {coNEXPTIME}$. We present an algorithm that computes the lexicographically optimal strategies via a reduction to computation of optimal strategies in a sequence of single-objectives games. We have implemented our algorithm and report experimental results on various case studies.

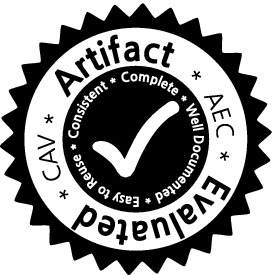



## Introduction

*Simple stochastic games (SGs)* [26] are zero-sum turn-based stochastic games played over a finite state space by two adversarial players, the Maximizer and Minimizer, along with randomness in the transition function. These games allow the interaction of angelic and demonic non-determinism as well as stochastic uncertainty. They generalize classical models such as Markov decision processes (MDPs) [39] which have only one player and stochastic uncertainty. An objective specifies a desired set of trajectories of the game, and the goal of the Maximizer is to maximize the probability of satisfying the objective against all choices of the Minimizer. The basic decision problem is to determine whether the Maximizer can ensure satisfaction of the objective with a given probability threshold. This problem is among the rare and intriguing combinatorial problems that are $\mathsf {NP}\cap \mathsf {coNP}$, and whether it belongs to P is a major and long-standing open problem. Besides the theoretical interest, SGs are a standard model in control and verification of stochastic reactive systems [4, 18, 31, 39], as well as they provide robust versions of MDPs when precise transition probabilities are not known [22, 45].

The multi-objective optimization problem is relevant in the analysis of systems with multiple, potentially conflicting goals, and a trade-off must be considered for the objectives. While the multi-objective optimization has been extensively studied for MDPs with various classes of objectives [1, 28, 39], the problem is notoriously hard for SGs. Even for multiple reachability objectives, such games are not determined [23] and their decidability is still open.

This work considers SGs with multiple reachability and safety objectives with lexicographic preference order over the objectives. That is, we consider SGs with several objectives where each objective is either reachability or safety, and there is a total preference order over the objectives. The motivation to study such lexicographic objectives is twofold. First, they provide an important special case of general multiple objectives. Second, lexicographic objectives are useful in many scenarios. For example, (i) an autonomus vehicle might have a primary objective to avoid clashes and a secondary objective to optimize performance; and (b) a robot saving lives during fire in a building might have a primary objective to save as many lives as possible, and a secondary objective to minimize energy consumption. Thus studying reactive systems with lexicographic objectives is a very relevant problem which has been considered in many different contexts [7, 33]. In particular non-stochastic games with lexicographic objectives [6, 25] and MDPs with lexicographic objectives [47] have been considered, but to the best of our knowledge SGs with lexicographic objectives have not been studied.

In this work we present several contributions for SGs with lexicographic reachability and safety objectives. The main contributions are as follows.

- *Determinacy.* In contrast to SGs with multiple objectives that are not determined, we establish determinacy of SGs with lexicographic combination of reachability and safety objectives.
- *Computational complexity.* For the associated decision problem we establish the following: (a) if the number of objectives is constant, then the decision problem lies in $\mathsf {NP}\cap \mathsf {coNP}$, matching the current known bound for SGs with a single objective; (b) in general the decision problem is $\mathsf {PSPACE}$-hard and can be solved in $\mathsf {NEXPTIME}\cap \mathsf {coNEXPTIME}$.
- *Strategy complexity.* We show that lexicographically optimal strategies exist that are deterministic but require finite memory. We also show that memory is only needed in order to remember the already satisfied and violated objectives.
- *Algorithm.* We present an algorithm that computes the unique lexicographic value and the witness lexicographically optimal strategies via a reduction to computation of optimal strategies in a sequence of single-objectives games.
- *Experimental results.* We have implemented the algorithm and present experimental results on several case studies.

*Technical Contribution.* The key idea is that, given the lexicographic order of the objectives, we can consider them sequentially. After every objective, we remove all actions that are not optimal, thereby forcing all following computation to consider only locally optimal actions. The main complication is that local optimality of actions does not imply global optimality when interleaving reachability and safety, as the latter objective can use locally optimal actions to stay in the safe region without reaching the more important target. We introduce quantified reachability objectives as a means to solve this problem.

*Related Work.* We present related works on: (a) MDPs with multiple objectives; (b) SGs with multiple objectives; (c) lexicographic objectives in related models; and (d) existing tool support.

- MDPs with multiple objectives have been widely studied over a long time [1, 39]. In the context of verifying MDPs with multiple objectives, both qualitative objectives such as reachability and LTL [29], as well as quantitative objectives, such as mean payoff [8, 13], discounted sum [17], or total reward [34] have been considered. Besides multiple objectives with expectation criterion, other criteria have also been considered, such as, combination with variance [9], or multiple percentile (threshold) queries [8, 20, 32, 41]. Practical applications of MDPs with multiple objectives are described in [2, 3, 42].
- More recently, SGs with multiple objectives have been considered, but the results are more limited [43]. Multiple mean-payoff objectives were first examined in [5] and the qualitative problems are coNP-complete [16]. Some special classes of SGs (namely stopping SGs) have been solved for total-reward objectives [23] and applied to autonomous driving [24]. However, even for the most basic question of solving SGs with multiple reachability objectives, decidability remains open.
- The study of lexicographic objectives has been considered in many different contexts [7, 33]. Non-stochastic games with lexicographic mean-payoff objectives and parity conditions have been studied in [6] for the synthesis of reactive systems with performance guarantees. Non-stochastic games with multiple $\omega$-regular objectives equipped with a monotonic preorder, which subsumes lexicographic order, have been studied in [12]. Moreover, the beyond worst-case analysis problems studied in [11] also considers primary and secondary objectives, which has a lexicographic flavor. MDPs with lexicographic discounted-sum objectives have been studied in [47], and have been extended with partial-observability in [46]. However, SGs with lexicographic reachability and safety objectives have not been considered so far.
- PRISM-Games [37] provides tool support for several multi-player multi- objective settings. MultiGain [10] is limited to generalized mean-payoff MDPs. Storm [27] can, among numerous single-objective problems, solve Markov automata with multiple timed reachability or expected cost objectives [40], multi-cost bounded reachability MDPs [35], and it can provide simple strategies for multiple expected reward objectives in MDPs [28].

*Structure of this Paper.* After recalling preliminaries and defining the problem in Sect. 2, we first consider games where all target sets are absorbing in Sect. 3. Then, in Sect. 4 we extend our insights to general games, yielding the full algorithm and the theoretical results. Finally, Sect. 5 describes the implementation and experimental evaluation. Section 6 concludes.

## Preliminaries

**Notation.** A probability distribution on a finite set *A* is a function $f: A \rightarrow [0,1]$ such that $\sum _{x\in A}f(x) =1$. We denote the set of all probability distributions on *A* by $\mathcal {D}(A)$. Vector-like objects $\textit{\textbf{x}}$ are denoted in a bold font and we use the notation $\textit{\textbf{x}}_i$ for the *i*-th component of $\textit{\textbf{x}}$. We use $\textit{\textbf{x}}_{<n}$ as a shorthand for $(\textit{\textbf{x}}_1,\ldots ,\textit{\textbf{x}}_{n-1}).$

### Basic Definitions

**Probabilistic Models.** In this paper, we consider *(simple) stochastic games* [26], which are defined as follows. Let $L= \{a,b,\ldots \}$ be a finite set of actions labels.

#### Definition 1

**(SG).** A *stochastic game* (SG) is a tuple $\mathcal {G}= (S_{\square },S_{\lozenge },\mathsf {Act}, P)$ with $S := S_{\square }\uplus S_{\lozenge }\ne \emptyset$ a finite set of states, $\mathsf {Act}: S \rightarrow 2^L\setminus \{\emptyset \}$ defines finitely many actions available at every state, and $P: S \times L\rightarrow \mathcal {D}(S)$ is the transition probability function. *P*(*s*, *a*) is undefined if $a \notin \mathsf {Act}(s)$.

We abbreviate $P(s,a)(s')$ to $P(s,a,s')$. We refer to the two players of the game as $\mathsf {Max}$ and $\mathsf {Min}$ and the sets $S_{\square }$ and $S_{\lozenge }$ are the $\mathsf {Max}$- and $\mathsf {Min}$-states, respectively. As the game is *turn based*, these sets partition the state space *S* such that in each state it is either $\mathsf {Max}$’s or $\mathsf {Min}$’s turn. The intuitive semantics of an SG is as follows: In every turn, the corresponding player picks one of the finitely many available actions $a \in \mathsf {Act}(s)$ in the current state *s*. The game then transitions to the next state according to the probability distribution *P*(*s*, *a*). The winning conditions are not part of the game itself and need to be further specified.

**Sinks, Markov Decision Processes and Markov Chains.** A state $s \in S$ is called *absorbing* (or sink) if $P(s,a,s) = 1$ for all $a \in \mathsf {Act}(s)$ and $\mathsf {Sinks}(\mathcal {G})$ denotes the set of all absorbing states of SG $\mathcal {G}$. A *Markov Decision Process* (MDP) is an SG where either $S_{\lozenge }= \emptyset$ or $S_{\square }= \emptyset$, i.e. a one-player game. A *Markov Chain* (MC) is an SG where $|\mathsf {Act}(s)|=1$ for all $s \in S$. For technical reasons, we allow countably infinite state spaces *S* for both MDPs and MCs.

**Strategies.** We define the formal semantics of games by means of *paths* and *strategies*. An *infinite path* $\pi$ is an infinite sequence $\pi = s_0 a_0 s_1 a_1 \dots \in (S \times L)^\omega$, such that for every $i \in \mathbb {N}$, $a_i\in \mathsf {Act}(s_i)$ and $s_{i+1} \in \{s' \mid P(s_i,a_i,s')>0\}$. *Finite path*s are defined analogously as elements of $(S \times L)^*\times S$. Note that when considering MCs, every state just has a single action, so an infinite path can be identified with an element of $S^\omega$.

A strategy of player $\mathsf {Max}$ is a function $\sigma :(S \times L)^* \times S_{\square }\rightarrow \mathcal {D}(L)$ where $\sigma (\pi s)(s')>0$ only if $s \in \mathsf {Act}(s)$. It is *memoryless* if $\sigma (\pi s) = \sigma (\pi ' s)$ for all $\pi , \pi ' \in (S \times L)^*$. More generally, $\sigma$ has memory of class-size at most *m* if the set $(S \times L)^*$ can be partitioned in *m* classes $M_1,\ldots ,M_m \subseteq (S \times L)^*$ such that $\sigma (\pi s) = \sigma (\pi ' s)$ for all $1 \le i \le m$, $\pi , \pi ' \in M_i$ and $s \in S_{\square }$. A memory of class-size *m* can be represented with $\lceil \log (m) \rceil$ bits.

A strategy is *deterministic* if $\sigma (\pi s)$ is Dirac for all $\pi s$. Strategies that are both memoryless and deterministic are called *MD* and can be identified as functions $\sigma :S_{\square }\rightarrow L$. Notice that there are at most $|L|^{S_{\square }}$ different MD strategies, that is, exponentially many in $S_{\square }$; in general, there can be uncountably many strategies.

Strategies $\tau$ of player $\mathsf {Min}$ are defined analogously, with $S_{\square }$ replaced by $S_{\lozenge }$. The set of all strategies of player $\mathsf {Max}$ is denoted with $\varSigma _{\mathsf {Max}}$, the set of all MD strategies with $\varSigma _{\mathsf {Max}}^{\mathsf {MD}}$, and similarly $\varSigma _{\mathsf {Min}}$ and $\varSigma _{\mathsf {Min}}^{\mathsf {MD}}$ for player $\mathsf {Min}$.

Fixing a strategy $\sigma$ of one player in a game $\mathcal {G}$ yields the *induced MDP* $\mathcal {G}^\sigma$. Fixing a strategy $\tau$ of the second player too, yields the *induced MC* $\mathcal {G}^{\sigma ,\tau }$. Notice that the induced models are finite if and only if the respective strategies use finite memory.

Given an (induced) MC $\mathcal {G}^{{\sigma ,\tau }}$, we let $\mathbb {P}_s^{\sigma ,\tau }$ be its associated probability measure on the Borel-measurable sets of infinite paths obtained from the standard cylinder construction where *s* is the initial state [39].

**Reachability and Safety.** In our setting, a *property* is a Borel-measurable set $\varOmega \subseteq S^\omega$ of infinite paths in an SG. The *reachability property* $\mathsf {Reach}\,\left( T\right)$ where $T \subseteq S$ is the set $\mathsf {Reach}\,\left( T\right) = \{s_0s_1\ldots \in S^\omega \mid \exists i \ge 0 :s_i \in T\}$. The set $\mathsf {Safe}\,\left( T\right) = S^\omega \setminus \mathsf {Reach}\,\left( T\right)$ is called a *safety property*. Further, for sets $T_1,T_2 \subseteq S$ we define the *until property* $T_1\ \mathsf {U}\ T_2 = \{s_0s_1\ldots \in S^\omega \mid \exists i \ge 0 :s_i \in T_2 \wedge \forall j < i :s_j \in T_1\}$. These properties are measurable (e.g. [4]). A reachability or safety property where the set *T* satisfies $T \subseteq \mathsf {Sinks}(\mathcal {G})$ is called *absorbing*. For the safety probabilities in an (induced) MC, it holds that $\mathbb {P}_s(\mathsf {Safe}\,\left( T\right) )= 1 - \mathbb {P}_s(\mathsf {Reach}\,\left( T\right) )$. We highlight that an objective $\mathsf {Safe}\,\left( T\right)$ is specified by the set of paths to avoid, i.e. paths satisfying the objective remain forever in $S \setminus T$.

### Stochastic Lexicographic Reachability-Safety Games

SGs with lexicographic preferences are a straightforward adaptation of the ideas of e.g. [46] to the game setting. The *lexicographic* order on $\mathbb {R}^n$ is defined as $\textit{\textbf{x}} \le _{\mathsf {lex}}\textit{\textbf{y}}$ iff $\textit{\textbf{x}}_i \le \textit{\textbf{y}}_i$ where $i \le n$ is the greatest position such that for all $j < i$ it holds that $\textit{\textbf{x}}_j = \textit{\textbf{y}}_j$. The position *i* thus acts like a *tiebreaker*. Notice that for arbitrary sets $X \subseteq [0,1]^n$, suprema and infima exist in the lexicographic order.

#### Definition 2

**(Lex-Objective and Lex-Value).** A *lexicographic reachability- safety objective* (*lex-objective*, for short) is a vector $\varvec{\varOmega }= (\varOmega _1,\ldots ,\varOmega _n)$ such that $\varOmega _i \in \{\mathsf {Reach}\,\left( S_i\right) , \mathsf {Safe}\,\left( S_i\right) \}$ with $S_i \subseteq S$ for all $1\le i \le n$. We call $\varvec{\varOmega }$ *absorbing* if all the $\varOmega _i$ are absorbing, i.e., if $S_i \subseteq \mathsf {Sinks}(\mathcal {G})$ for all $1 \le i \le n$. The *lex-(icographic)value* of $\varvec{\varOmega }$ at state $s \in S$ is defined as:

1 $$\begin{aligned} ^{\varvec{\varOmega }}\mathbf {v}^{\mathsf {lex}}(s) = \sup _{\sigma \in \varSigma _{\mathsf {Max}}} \inf _{\tau \in \varSigma _{\mathsf {Min}}} \mathbb {P}^{\sigma ,\tau }_s(\varvec{\varOmega }) \end{aligned}$$

where $\mathbb {P}^{\sigma ,\tau }_s(\varvec{\varOmega })$ denotes the *vector* $(\mathbb {P}^{\sigma ,\tau }_s(\varOmega _1),\ldots ,\mathbb {P}^{\sigma ,\tau }_s(\varOmega _n))$ and the suprema and infima are taken with respect to the order $\le _{\mathsf {lex}}$ on $[0,1]^n$.

Thus the lex-value at state *s* is the lexicographically supremal vector of probabilities that $\mathsf {Max}$ can ensure against all possible behaviors of $\mathsf {Min}$. We will prove in Sect. 4.3 that the supremum and infimum in (1) can be exchanged; this property is called *determinacy*. We omit the superscript $\varvec{\varOmega }$ in $^{\varvec{\varOmega }}\mathbf {v}^{\mathsf {lex}}$ if it is clear from the context. We also omit the sets $\varSigma _{\mathsf {Max}}$ and $\varSigma _{\mathsf {Min}}$ in the suprema in (1), e.g. we will just write $\sup _{\sigma }$.

**Fig. 1.**
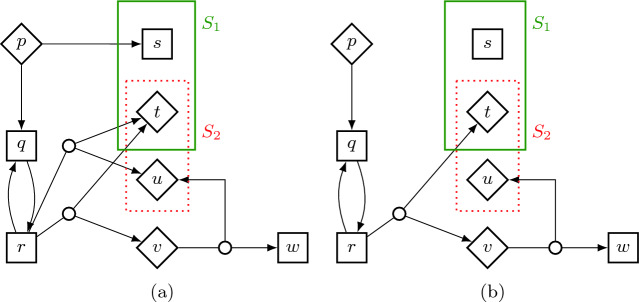
(a) An example of a stochastic game. $\mathsf {Max}$-states are rendered as squares $\square$ and $\mathsf {Min}$-states as rhombs $\lozenge$. Probabilistic choices are indicated with small circles. In this example, all probabilities equal . The absorbing lex-objective $\varvec{\varOmega }= \{\mathsf {Reach}\,\left( S_1\right) , \mathsf {Safe}\,\left( S_2\right) \}$ is indicated by the thick green line around $S_1 = \{s,t\}$ and the dotted red line around $S_2 = \{t,u\}$. Self-loops in sinks are omitted. (b) Restriction of the game to lex-optimal actions only.

#### Example 1

*(SGs and lex-values).* Consider the SG sketched in Fig. 1a with the lex-objective $\varvec{\varOmega }= \{\mathsf {Reach}\,\left( S_1\right) , \mathsf {Safe}\,\left( S_2\right) \}$. Player $\mathsf {Max}$ must thus maximize the probability to reach $S_1$ and, moreover, among all possible strategies that do so, it must choose one that maximizes the probability to avoid $S_2$ forever. $\triangle$

**Lex-Value of Actions and Lex-Optimal Actions.** We extend the notion of value to actions. Let $s \in S$ be a state. The *lex-value of an action* $a \in \mathsf {Act}(s)$ is defined as $\mathbf {v}^{\mathsf {lex}}(s,a) = \sum _{s'}P(s,a,s')\mathbf {v}^{\mathsf {lex}}(s')$. If $s \in S_{\square }$, then action *a* is called *lex-optimal* if $\mathbf {v}^{\mathsf {lex}}(s,a) = \max _{b \in \mathsf {Act}(s)}\mathbf {v}^{\mathsf {lex}}(s,b)$. Lex-optimal actions are defined analogously for states $s \in S_{\lozenge }$ by considering the minimum instead of the maximum. Notice that there is always at least one optimal action because $\mathsf {Act}(s)$ is finite by definition.

#### Example 2

*(Lex-value of actions).* We now intuitively explain the lex-values of all states in Fig. 1a. The lex-value of sink states *s*, *t*, *u* and *w* is determined by their membership in the sets $S_1$ and $S_2$. E.g., $\mathbf {v}^{\mathsf {lex}}(s) = (1,1)$, as it is part of the set $S_1$ that should be reached and not part of the set $S_2$ that should be avoided. Similarly we get the lex-values of *t*, *u* and *w* as (1, 0), (0, 0) and (0, 1) respectively. State *v* has a single action that yields (0, 0) or (0, 1) each with probability , thus .

State *p* has one action going to *s*, which would yield (1, 1). However, as *p* is a $\mathsf {Min}$-state, its best strategy is to avoid giving such a high value. Thus, it uses the action going downwards and $\mathbf {v}^{\mathsf {lex}}(p)=\mathbf {v}^{\mathsf {lex}}(q)$. State *q* only has a single action going to *r*, so $\mathbf {v}^{\mathsf {lex}}(q)=\mathbf {v}^{\mathsf {lex}}(r)$.

State *r* has three choices: (i) Going back to *q*, which results in an infinite loop between *q* and *r*, and thus never reaches $S_1$. So a strategy that commits to this action will not achieve the optimal value. (ii) Going to *t* or *u* each with probability . In this case, the safety objective is definitely violated, but the reachability objective achieved with . (iii) Going to *t* or *v* each with probability . Similarly to (ii), the probability to reach $S_1$ is , but additionally, there is a  chance to avoid $S_2$. Thus, since *r* is a $\mathsf {Max}$-state, its lex-optimal choice is the action leading to *t* or *v* and we get . $\triangle$

Notice that with the kind of objectives considered, we can easily swap the roles of $\mathsf {Max}$ and $\mathsf {Min}$ by exchanging safety objectives with reachability and vice versa. It is thus no loss of generality to consider subsequently introduced notions such as optimal strategies only from the perspective of $\mathsf {Max}$.

#### Definition 3

**(Lex-Optimal Strategies).** A strategy $\sigma \in \varSigma _{\mathsf {Max}}$ is *lex-optimal* for $\varvec{\varOmega }$ if for all $s \in S$, $\mathbf {v}^{\mathsf {lex}}(s) = \inf _{\tau '} \mathbb {P}_s^{\sigma ,\tau '}(\varvec{\varOmega })$. A strategy $\tau$ of $\mathsf {Min}$ is a *lex-optimal counter-strategy* against $\sigma$ if $\mathbb {P}_s^{\sigma ,\tau }(\varvec{\varOmega }) = \inf _{\tau '} \mathbb {P}_s^{\sigma ,\tau '}(\varvec{\varOmega })$.

We stress that counter-strategies of $\mathsf {Min}$ depend on the strategy chosen by $\mathsf {Max}$.

**Locally Lex-Optimal Strategies.** An MD strategy $\sigma$ of $\mathsf {Max}$ ($\mathsf {Min}$, resp.) is called *locally lex-optimal* if for all $s \in S_{\square }$ ($s \in S_{\lozenge }$, resp.) and $a \in \mathsf {Act}(s)$, we have $\sigma (s)(a) > 0$ implies that action *a* is lex-optimal. Thus, locally lex-optimal strategies only assign positive probability to lex-optimal actions.

**Convention.** For the rest of the paper, unless stated otherwise, we use $\mathcal {G}= (S_{\square },S_{\lozenge },\mathsf {Act},P)$ to denote an SG and $\varvec{\varOmega }=(\varOmega _1,\ldots ,\varOmega _n)$ is a suitable (not necessarily absorbing) lex-objective, that is $\varOmega _i \in \{\mathsf {Reach}\,\left( S_i\right) , \mathsf {Safe}\,\left( S_i\right) \}$ with $S_i \subseteq S$ for all $1 \le i \le n$.

## Lexicographic SGs with Absorbing Targets

In this section, we show how to compute the lexicographic value for SGs where *all target sets are absorbing*. We first show various theoretical results in Sect. 3.1 upon which the algorithm for computing the values and optimal strategies presented in Sect. 3.2 is then built. The main technical difficulty arises from interleaving reachability and safety objectives. In Sect. 4, we will reduce solving general (not necessarily absorbing) SGs to the case with absorbing targets.

### Characterizing Optimal Strategies

This first subsection derives a characterization of lex-optimal strategies in terms of local optimality and an additional reachability condition (Lemma 2 further below). It is one of the key ingredients for the correctness of the algorithm presented later and also gives rise to a (non-constructive) proof of existence of MD lex-optimal strategies in the absorbing case.

We begin with the following lemma that summarizes some straightforward facts we will frequently use. Recall that a strategy is *locally lex-optimal* if it only selects actions with optimal lex-value.

#### Lemma 1

The following statements hold for any absorbing lex-objective $\varvec{\varOmega }$:

- If $\sigma \in \varSigma _{\mathsf {Max}}^{\mathsf {MD}}$ is lex-optimal and $\tau \in \varSigma _{\mathsf {Min}}^{\mathsf {MD}}$ is a lex-optimal counter strategy against $\sigma$, then $\sigma$ and $\tau$ are both *locally* lex-optimal. (We do not yet claim that such strategies ${\sigma ,\tau }$ always exist.)
- Let $\widetilde{\mathcal {G}}$ be obtained from $\mathcal {G}$ by removing all actions (of both players) that are not locally lex-optimal. Let $\widetilde{\mathbf {v}}^\mathsf {lex}$ be the lex-values in $\widetilde{\mathcal {G}}$. Then $\widetilde{\mathbf {v}}^\mathsf {lex}= \mathbf {v}^{\mathsf {lex}}$.

#### Proof

*(Sketch).* Both claims follow from the definitions of lex-value and lex-optimal strategy. For (b) in particular, we show that a strategy using actions which are not lex-optimal can be transformed into a strategy that achieves a greater (lower, resp.) value. Thus removing the non lex-optimal actions does not affect the lex-value. See [19, Appendix A.1] for more technical details.    $\square$

#### Example 3

*(Modified game* $\widetilde{\mathcal {G}}$*).* Consider again the SG from Fig. 1a. Recall the lex-values from Example 1. Now we remove the actions that are not locally lex-optimal. This means we drop the action that leads from *p* to *s* and the action that leads from *r* to *t* or *u* (Fig. 1b). Since these actions were not used by the lex-optimal strategies, the value in the modified SG is the same as that of the original game. $\triangle$

#### Example 4

*(Locally lex-optimal does not imply globally lex-optimal).* Note that we do not drop the action that leads from *r* to *q*, because $\mathbf {v}^{\mathsf {lex}}(r)=\mathbf {v}^{\mathsf {lex}}(q)$, so this action is locally lex-optimal. In fact, a lex-optimal strategy can use it arbitrarily many times without reducing the lex-value, as long as eventually it picks the action leading to *t* or *v*. However, if we only played the action leading to *q*, the lex-value would be reduced to (0, 1) as we would not reach $S_1$, but would also avoid $S_2$.

We stress the following consequence of this: Playing a locally lex-optimal strategy is not necessarily globally lex-optimal. It is not sufficient to just restrict the game to locally lex-optimal actions of the previous objectives and then solve the current one. Note that in fact the optimal strategy for the second objective $\mathsf {Safe}\,\left( S_2\right)$ would be to remain in $\{p,q\}$; however, we must not pick this safety strategy, before we have not “tried everything” for all previous reachability objectives, in this case reaching $S_1$. $\triangle$

This idea of “trying everything” for an objective $\mathsf {Reach}\,\left( S_i\right)$ is equivalent to the following: either reach the target set $S_i$, or reach a set of states from which $S_i$ cannot be reached anymore. Formally, let $\mathsf {Zero}_i = \{ s \in S \mid \mathbf {v}^{\mathsf {lex}}_i(s) = 0 \}$ be the set of states that cannot reach the target set $S_i$ anymore. Note that it depends on the lex-value, not the single-objective value. This is important, as the single-objective value could be greater than 0, but a more important objective has to be sacrificed to achieve it.

We define the set of states where we have “tried everything” for all reachability objectives as follows:

#### Definition 4

**(Final Set).** For absorbing $\varvec{\varOmega }$, let $R_{<i} = \{j<i \mid \varOmega _j = \mathsf {Reach}\,\left( S_j\right) \}$. We define the *final set* $F_{<i} = \bigcup _{k \in R_{<i}} S_k\ \cup \ \bigcap _{k\in R_{<i}}\mathsf {Zero}_k$ with the convention that $F_{<i} = S$ if $R_{<i} = \emptyset$. We also let $F = F_{<n+1}$.

The final set contains all target states as well as the states that have lex-value 0 for all reachability objectives; we need the intersection of the sets $\mathsf {Zero}_k$, because as long as a state still has a positive probability to reach any target set, its optimal behaviour is to try that.

#### Example 5

*(Final set).* For the game in Fig. 1, we have $\mathsf {Zero}_1 = \{u,v,w\}$ and thus $F = \mathsf {Zero}_1 \cup S_1 = \{s,t,u,v,w\}$. An MD lex-optimal strategy of $\mathsf {Max}$ must almost-surely reach this set against any strategy of $\mathsf {Min}$; only then it has “tried everything”. $\triangle$

The following lemma characterizes MD lex-optimal strategies in terms of local lex-optimality and the final set.

#### Lemma 2

Let $\varvec{\varOmega }$ be an absorbing lex-objective and $\sigma \in \varSigma _{\mathsf {Max}}^{\mathsf {MD}}$. Then $\sigma$ is lex-optimal for $\varvec{\varOmega }$ if and only if $\sigma$ is locally lex-optimal and for all $s \in S$ we have

$$\begin{aligned} \forall \tau \in \varSigma _{\mathsf {Min}}^{\mathsf {MD}}:\mathbb {P}_s^{\sigma ,\tau }(\mathsf {Reach}\,\left( F\right) ) = 1. \qquad \qquad \qquad \qquad (\star ) \end{aligned}$$

#### Proof

*(Sketch).* The “*if*”-direction is shown by induction on the number *n* of targets. We make a case distinction according to the type of $\varOmega _n$: If it is safety, then we prove that local lex-optimality is already sufficient for global lex-optimality. Else if $\varOmega _n$ is reachability, then intuitively, the additional condition ($\star$) ensures that the strategy $\sigma$ indeed “tries everything” and either reaches the target $S_n$ or eventually a state in $\mathsf {Zero}_n$ where the opponent $\mathsf {Min}$ can make sure that $\mathsf {Max}$ cannot escape. The technical details of these assertions rely on a fixpoint characterization of the reachability probabilities combined with the classic Knaster-Tarski Fixpoint Theorem [44] and are given in [19, Appendix A.2].

For the “*only if*”-direction recall that lex-optimal strategies are necessarily locally lex-optimal by Lemma 1 (a). Further let *i* be such that $\varOmega _i = \mathsf {Reach}\,\left( S_i\right)$ and assume for contradiction that $\sigma$ remains forever within $S \setminus (S_i \cup \mathsf {Zero}_i)$ with positive probability against some strategy of $\mathsf {Min}$. But then $\sigma$ visits states with positive lex-value for $\varOmega _i$ infinitely often without ever reaching $S_i$. Thus $\sigma$ is not lex-optimal, contradiction.    $\square$

Finally, this characterization allows us to prove that MD lex-optimal strategies exist for absorbing objectives.

#### Theorem 1

For an absorbing lex-objective $\varvec{\varOmega }$, there exist MD lex-optimal strategies for both players.

#### Proof

*(Sketch).* We consider the subgame $\widetilde{\mathcal {G}}$ obtained by removing lex-sub-optimal actions for both players and then show that the (single-objective) value of $\mathsf {Reach}\,\left( F\right)$ in $\widetilde{\mathcal {G}}$ equals 1. An optimal MD strategy for $\mathsf {Reach}\,\left( F\right)$ exists [26]; further, it is locally lex-optimal, because we are in $\widetilde{\mathcal {G}}$, and it reaches *F* almost surely. Thus, it is lex-optimal for $\varvec{\varOmega }$ by the “*if*”-direction of Lemma 2. See [19, Appendix A.3] for more details on the proof.    $\square$

### Algorithm for SGs with Absorbing Targets

Theorem 1 is not constructive because it relies on the values $\mathbf {v}^{\mathsf {lex}}$ without showing how to compute them. Computing the values and constructing an optimal strategy for $\mathsf {Max}$ in the case of an absorbing lex-objective is the topic of this subsection.

#### Definition 5

**(QRO).** A *quantified reachability objective* (QRO) is determined by a function $q:S' \rightarrow [0,1]$ where $S' \subseteq S$. For all strategies $\sigma$ and $\tau$, we define:

$$ \mathbb {P}^{{\sigma ,\tau }}_s(\mathsf {Reach}\,\left( q\right) ) = \sum _{t \in S'} \mathbb {P}^{{\sigma ,\tau }}_s((S \setminus S')\ \mathsf {U}\ t) \cdot q(t). $$

Intuitively, a QRO generalizes its standard Boolean counterpart by additionally assigning a weight to the states in the target set $S'$. Thus the probability of a QRO is obtained by computing the sum of the $q(t)$, $t \in S'$, weighted by the probability to avoid $S'$ until reaching *t*. Note that this probability does not depend on what happens after reaching $S'$; so it is unaffected by making all states in $S'$ absorbing.

In Sect. 4, we need the dual notion of a quantified safety property, defined as $\mathbb {P}^{{\sigma ,\tau }}_s(\mathsf {Safe}\,\left( q\right) ) = 1 - \mathbb {P}^{{\sigma ,\tau }}_s(\mathsf {Reach}\,\left( q\right) )$; intuitively, this amounts to minimizing the reachability probability.

#### Remark 1

A usual reachability property $\mathsf {Reach}\,\left( S'\right)$ is a special case of a quantified one with $q(s) = 1$ for all $s \in S'$. Vice versa, quantified properties can be easily reduced to usual ones defined only by the set $S'$: Convert all states $t \in S'$ into sinks, then for each such *t* prepend a new state $t'$ with a single action *a* and $P(t',a,t)=q(t)$ and $P(t',a,\bot )=1-q(t)$ where $\bot$ is a sink state. Finally, redirect all transitions leading into *t* to $t'$. Despite this equivalence, it turns out to be convenient and natural to use QROs.

#### Example 6

*(QRO).* Example 4 illustrated that solving a safety objective after a reachability objective can lead to problems, as the optimal strategy for $\mathsf {Safe}\,\left( S_2\right)$ did not use the action that actually reached $S_1$. In Example 5 we indicated that the final set $F = \{s,t,u,v,w\}$ has to be reached almost surely, and among those states the ones with the highest safety values should be preferred. This can be encoded in a QRO as follows: Compute the values for the $\mathsf {Safe}\,\left( S_2\right)$ objective for the states in *F*. Then construct the function $q_2 :F \rightarrow [0,1]$ that maps all states in *F* to their safety value, i.e., . $\triangle$

Thus using QROs, we can effectively reduce (interleaved) safety objectives to quantified *reachability* objectives:

#### Lemma 3

**(Reduction Safe** $\rightarrow$ **Reach).** Let $\varvec{\varOmega }$ be an absorbing lex-objective with $\varOmega _n = \mathsf {Safe}\,\left( S_n\right)$, $q_n :F \rightarrow [0,1]$ with $q_n(t) = \mathbf {v}^{\mathsf {lex}}_n(t)$ for all $t \in F$ where *F* is the final set (Definition 4), and $\varvec{\varOmega }'=(\varOmega _1,\ldots ,\varOmega _{n-1},\mathsf {Reach}\,\left( q_n\right) )$. Then: $^{\varvec{\varOmega }}\mathbf {v}^{\mathsf {lex}}~=~^{\varvec{\varOmega }'}\mathbf {v}^{\mathsf {lex}}$.

#### Proof

*(Sketch).* By definition, $^{\varvec{\varOmega }}\mathbf {v}^{\mathsf {lex}}(s)~=~^{\varvec{\varOmega }'}\mathbf {v}^{\mathsf {lex}}(s)$ for all $s \in F$, so we only need to consider the states in $S \setminus F$. Since any lex-optimal strategy for $\varvec{\varOmega }$ or $\varvec{\varOmega }'$ must also be lex-optimal for $\varvec{\varOmega }_{<n}$, we know by Lemma 2 that such a strategy reaches $F_{<n}$ almost-surely. Note that we have $F_{<n} = F$, as the *n*-th objective, either the QRO or the safety objective, does not add any new states to *F*. The reachability objective $\mathsf {Reach}\,\left( q_n\right)$ weighs the states in *F* with their lexicographic safety values $\mathbf {v}^{\mathsf {lex}}_n$. Thus we additionally ensure that in order to reach *F*, we use those actions that give us the best safety probability afterwards. In this way we obtain the correct lex-values $\mathbf {v}^{\mathsf {lex}}_n$ even for states in $S \setminus F$. See [19, Appendix A.4] for the full technical proof.    $\square$

#### Example 7

*(Reduction Safe* $\rightarrow$ *Reach).* Recall Example 6. By the preceding Lemma 3, computing $\sup _{\sigma } \inf _{\tau } \mathbb {P}^{{\sigma ,\tau }}_s(\mathsf {Reach}\,\left( S_1\right) , \mathsf {Reach}\,\left( q_2\right) )$ yields the correct lex-value $\mathbf {v}^{\mathsf {lex}}(s)$ for all $s \in S$. Consider for instance state *r* in the running example: The action leading to *q* is clearly suboptimal for $\mathsf {Reach}\,\left( q_2\right)$ as it does not reach *F*. Both other actions surely reach *F*. However, since $q_2(t) = q_2(u) = 0$ while $q_2(v) = \nicefrac 1 2$, the action leading to *u* and *v* is preferred over that leading to *t* and *u*, as it ensures the higher safety probability after reaching *F*. $\triangle$


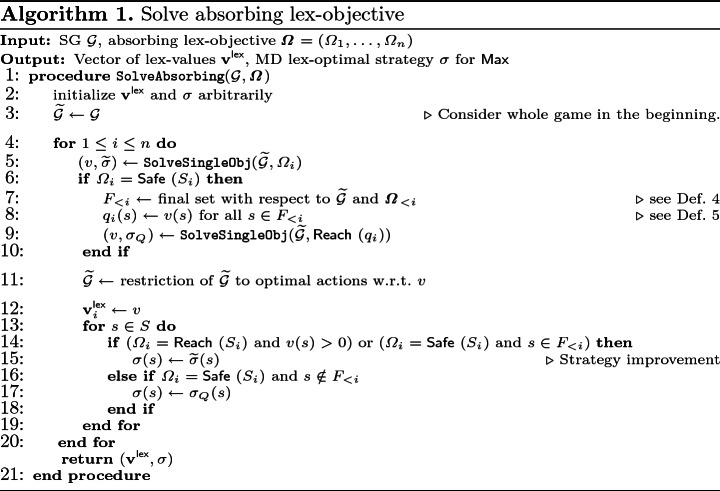


We now explain the basic structure of Algorithm 1. More technical details are explained in the proof sketch of Theorem 2 and the full proof is in [19, Appendix A.5]. The idea of Algorithm 1 is, as sketched in Sect. 3.1, to consider the objectives sequentially in the order of importance, i.e., starting with $\varOmega _1$. The *i*-th objective is solved (Lines 5–10) and the game is restricted to only the locally optimal actions (Line 11). This way, in the *i*-th iteration of the main loop, only actions that are locally lex-optimal for objectives 1 through $(i{-}1)$ are considered. Finally, we construct the optimal strategy and update the result variables (Lines 12–19).

#### Theorem 2

Given an SG $\mathcal {G}$ and an absorbing lex-objective $\varvec{\varOmega }= (\varOmega _1, \dots , \varOmega _n)$, Algorithm 1 correctly computes the vector of lex-values $\mathbf {v}^{\mathsf {lex}}$ and an MD lex-optimal strategy $\sigma$ for player $\mathsf {Max}$. It needs *n* calls to a single objective solver.

#### Proof

*(Sketch).*

- $\widetilde{\mathcal {G}}$**-invariant:** For $i>1$, in the *i*-th iteration of the loop, $\widetilde{\mathcal {G}}$ is the original SG restricted to only those actions that are locally lex-optimal for the targets 1 to $(i{-}1)$; this is the case because Line 11 was executed for all previous targets.
- **Single-objective case:** The single-objective that is solved in Line 5 can be either reachability or safety. We can use any (precise) single-objective solver as a black box, e.g. strategy iteration [36]. Recall that by Remark 1, it is no problem to call a single-objective solver with a QRO since there is a trivial reduction.
- **QRO for safety:** If an objective is of type reachability, no further steps need to be taken; if on the other hand it is safety, we need to ensure that the problem explained in Example 4 does not occur. Thus we compute the final set $F_{<i}$ for the *i*-th target and then construct and solve the QRO as in Lemma 3.
- **Resulting strategy:** When storing the resulting strategy, we again need to avoid errors induced by the fact that locally lex-optimal actions need not be globally lex-optimal. This is why for a reachability objective, we only update the strategy in states that have a positive value for the current objective; if the value is 0, the current strategy does not have any preference, and we need to keep the old strategy. For safety objectives, we need to update the strategy in two ways: for all states in the final set $F_{<i}$, we set it to the safety strategy $\widetilde{\sigma }$ (from Line 5) as within $F_{<i}$ we do not have to consider the previous reachability objectives and therefore must follow an optimal safety strategy. For all states in $S \setminus F_{<i}$, we set it to the reachability strategy from the QRO $\sigma _Q$ (from Line9). This is correct, as $\sigma _Q$ ensures almost-sure reachability of $F_{<i}$ which is necessary to satisfy all preceding reachability objectives; moreover $\sigma _Q$ prefers those states in $F_{<i}$ that have a higher safety value (cf. Lemma 3).
- **Termination:** The main loop of the algorithm invokes $\mathtt {SolveSingleObj}$ for each of the *n* objectives.

   $$\square $$

## General Lexicographic SGs

We now consider $\varvec{\varOmega }$ where $S_i \subseteq \mathsf {Sinks}(\mathcal {G})$ does *not* necessarily hold. Section 4.1 describes how we can reduce these general lex-objectives to the absorbing case. The resulting algorithm is given in Sect. 4.2 and the theoretical implications in Sect. 4.3.

### Reducing General Lexicographic SGs to SGs with Absorbing Targets

In general lexicographic SG, strategies need memory, because they need to remember which of the $S_i$ have already been visited and behave accordingly. We formalize the solution of such games by means of *stages*. Intuitively, one can think of a stage as a copy of the game with less objectives, or as the sub-game that is played after visiting some previously unseen set $S_i$.

#### Definition 6

**(Stage).** Given an arbitrary lex-objective $\varvec{\varOmega }= (\varvec{\varOmega }_1, \dots , \varvec{\varOmega }_n)$ and a set $I \subseteq \{i \le n\}$, a *stage* $\varvec{\varOmega }(I)$ is the objective vector where the objectives $\varvec{\varOmega }_i$ are removed for all $i \in I$.

For state $s \in S$, let $\varvec{\varOmega }(s)=\varvec{\varOmega }(\{i \mid s \in S_i\})$. If a stage contains only one objective, we call it *simple*.

**Fig. 2.**
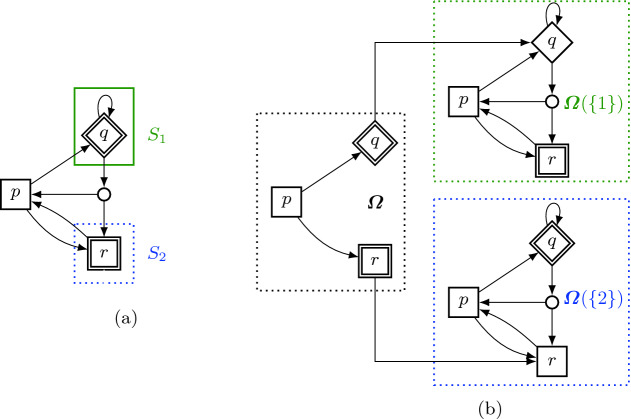
(a) SG with non-absorbing lex-objective $\varvec{\varOmega }= (\mathsf {Reach}\,\left( S_1\right) , \mathsf {Reach}\,\left( S_2\right) )$. (b) The three stages identified by the sub-objectives $\varvec{\varOmega }$, $\varvec{\varOmega }(\{1\}) = (\mathsf {Reach}\,\left( S_2\right) )$ and $\varvec{\varOmega }(\{2\}) = (\mathsf {Reach}\,\left( S_1\right) )$. The two stages on the right are both *simple*.

#### Example 8

*(Stages).* Consider the SG in Fig. 2a. As there are two objectives, there are four possible stages: The one where we consider both objectives (the region denoted with $\varvec{\varOmega }$ in Fig. 2b), the *simple* ones where we consider only one of the objectives (regions $\varvec{\varOmega }(\{1\})$ and $\varvec{\varOmega }(\{2\})$), and the one where both objectives have been visited. The last stage is trivial since there are no more objectives, hence we do not depict it and do not have to consider it. The actions of *q* and *r* are omitted in the $\varvec{\varOmega }$-stage, as upon visiting these states, a new stage begins.

Consider the simple stages: in stage $\varvec{\varOmega }(\{1\})$, *q* has value 0, as it is a $\mathsf {Min}$-state and will use the self-loop to avoid reaching $r \in S_2$. In stage $\varvec{\varOmega }(\{2\})$, both *p* and *r* have value 1, as they can just go to the target state $q \in S_1$. Combining this knowledge, we can get an optimal strategy for every state. In particular, note that an optimal strategy for state *p* needs memory: First go to *r* and thereby reach stage $\varvec{\varOmega }(\{2\})$. Afterwards, go from *r* to *p* and now, on the second visit in a different stage, use the other action in *p* to reach *q*. In this example, we observe another interesting fact about lexicographic games: it can be optimal to first satisfy less important objectives. $\triangle$

In the example, we combined our knowledge of the sub-stages to find the lex-values for the whole lex-objective. In general, the values for the stages are numbers in [0, 1]. Thus we reuse the idea of *quantified* reachability and safety objectives, see Definition 5.

For all $1 \le i \le n$, let $q_i :\bigcup _{j\le n} S_j \rightarrow [0,1]$ by defined by:To keep the correct type of every objective, we let $\mathsf {q}\varvec{\varOmega }= (\mathsf {type}_1(q_1),\ldots ,\mathsf {type}_n(q_n))$ where for all $1 \le i \le n$, $\mathsf {type}_i = \mathsf {Reach}$ if $\varOmega _i=\mathsf {Reach}\,\left( S_i\right)$ and else $\mathsf {type}_i = \mathsf {Safe}$ if $\varOmega _i = \mathsf {Safe}\,\left( S_i\right)$. So we have now reduced a general lexicographic objective $\varvec{\varOmega }$ to a vector of quantitative objectives $\mathsf {q}\varvec{\varOmega }$. Lemma 4 shows that this reduction preserves the values.

#### Lemma 4

For arbitrary lex-objectives $\varvec{\varOmega }$ it holds that $^{\varvec{\varOmega }}\mathbf {v}^{\mathsf {lex}}=\ ^{\mathsf {q}\varvec{\varOmega }}\mathbf {v}^{\mathsf {lex}}$.

#### Proof

*(Sketch).* We write $\mathfrak {S}= \bigcup _{j\le n} S_j$ for the sake of readability in this sketch. By induction on the length *n* of the lex-objective $\varvec{\varOmega }$, it is easy to show that the equation holds in states $s \in \mathfrak {S}$, i.e., $^{\varvec{\varOmega }}\mathbf {v}^{\mathsf {lex}}(s) =\ ^{\mathsf {q}\varvec{\varOmega }}\mathbf {v}^{\mathsf {lex}}(s)$. For a state *s* which is not contained in any of the $S_j$, and for any strategies ${\sigma ,\tau }$ we have the following equation

$$\begin{aligned} \mathbb {P}^{\sigma ,\tau }_s(\mathsf {Reach}\,\left( S_i\right) )&= \sum _{\pi t \in Paths_{fin}(\mathfrak {S})} \mathbb {P}_s^{\sigma ,\tau }(\pi t) \cdot \mathbb {P}_{\pi t}^{{\sigma ,\tau }}(\mathsf {Reach}\,\left( S_i\right) ) \end{aligned}$$

where $Paths_{fin}(\mathfrak {S}) = \{\pi t \in ((S \setminus \mathfrak {S})\times L)^* \times S \mid t \in \mathfrak {S}\}$ denotes the set of all finite paths to a state in $\mathfrak {S}$ in the Markov chain $\mathcal {G}^{\sigma ,\tau }$ and $\mathbb {P}_s^{\sigma ,\tau }(\pi t)$ is the probability of such a path when $\mathcal {G}^{\sigma ,\tau }$ starts in *s*. From this we deduce that in order to maximize the left hand size of the equation in the lexicographic order, we should play such that we prefer reaching states in $\mathfrak {S}$ where $q_i$ has a higher value; that is, we should maximize the QRO $\mathsf {Reach}\,\left( q_i\right)$. The argument for safety is similar and detailed in [19, Appendix A.6].    $\square$

The functions $q_i$ involved in $\mathsf {q}\varvec{\varOmega }$ *all have the same domain* $\bigcup _{j\le n} S_j$. Hence we can, as mentioned below Definition 5, consider $\mathsf {q}\varvec{\varOmega }$ on the game where all states in $\bigcup _{j\le n} S_j$ are sinks without changing the lex-value. This is precisely the definition of an absorbing game, and hence we can compute $^{\mathsf {q}\varvec{\varOmega }}\mathbf {v}^{\mathsf {lex}}$ using Algorithm 1 from Sect. 3.2.

### Algorithm for General SG

Algorithm 2 computes the lex-value $^{\varvec{\varOmega }}\mathbf {v}^{\mathsf {lex}}$ for a given lexicographic objective $\varvec{\varOmega }$ and an arbitrary SG $\mathcal {G}$. We highlight the following technical details:

- **Reduction to absorbing case:** We just have seen, that once we have the quantitative objective vector $\mathsf {q}\varvec{\varOmega }$, we can use the algorithm for absorbing SG (Line 12).
- **Computing the quantitative objective vector:** To compute $\mathsf {q}\varvec{\varOmega }$, the algorithm calls itself recursively on all states in the union of all target sets (Line 5–7). We annotated this recursive call “With dynamic programming”, as we can reuse the results of the computations. In the worst case, we have to solve all $2^n - 1$ possible non-empty stages. Finally, given the values $^{\varvec{\varOmega }(s)}\mathbf {v}^{\mathsf {lex}}$ for all $s \in \bigcup _{j\le n} S_j$, we can construct the quantitative objective (Line 9 and 11) that is used for the call to $\mathtt {SolveAbsorbing}$.
- **Termination:** Since there are finitely many objectives in $\varvec{\varOmega }$ and in every recursive call at least one objective is removed from consideration, eventually we have a *simple* objective that can be solved by $\mathtt {SolveSingleObj}$ (Line 3).
- **Resulting strategy:** The resulting strategy is composed in Line 13: It adheres to the strategy for the quantitative query $^{\mathsf {q}\varvec{\varOmega }}\sigma$ until some $s \in \bigcup _{j \le n} S_j$ is reached. Then, to achieve the values promised by $q_i(s)$ for all *i* with $s \notin S_i$, it adheres to $^{\varvec{\varOmega }(s)} \sigma$, the optimal strategy for stage $\varvec{\varOmega }(s)$ obtained by the recursive call.

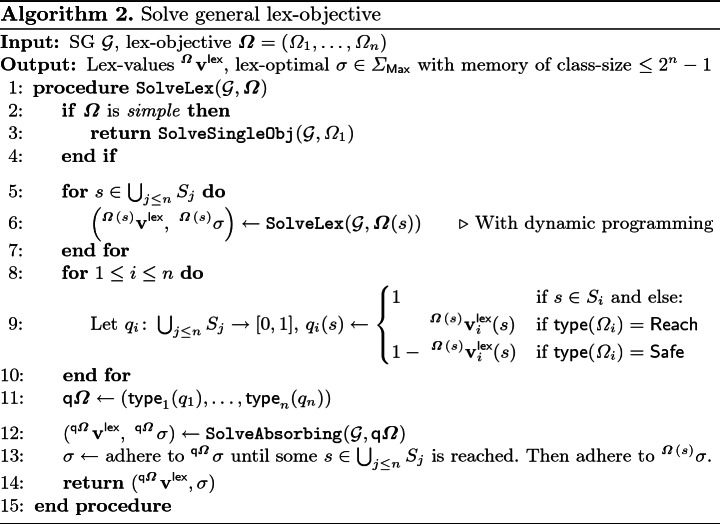


#### Corollary 1

Given an SG $\mathcal {G}$ and an arbitrary lex-objective $\varvec{\varOmega }= (\varOmega _1,\dots ,\varOmega _n)$, Algorithm 2 correctly computes the vector of lex-values $\mathbf {v}^{\mathsf {lex}}$ and a deterministic lex-optimal strategy $\sigma$ of player $\mathsf {Max}$ which uses memory of class-size $\le 2^n -1$. The algorithm needs at most $2^n -1$ calls to $\mathtt {SolveAbsorbing}$ or $\mathtt {SolveSingleObj}$.

#### Proof

Correctness of the algorithm and termination follows from the discussion of the algorithm, Lemma 4 and Theorem 2.    $\square$

### Theoretical Implications: Determinacy and Complexity

Theorem 3 below states that lexicographic games are *determined* for arbitrary lex-objectives $\varvec{\varOmega }$. Intuitively, this means that the lex-value is independent from the player who fixes their strategy first. Recall that this property does not hold for non-lexicographic multi-reachability/safety objectives [23].

#### Theorem 3

**(Determinacy).** For general SG $\mathcal {G}$ and lex-objective $\varvec{\varOmega }$, it holds for all $s \in S$ that:

$$ \mathbf {v}^{\mathsf {lex}}(s) = \sup _{\sigma } \inf _{\tau } \mathbb {P}_s^{\sigma ,\tau }(\varvec{\varOmega }) = \inf _{\tau } \sup _{\sigma } \mathbb {P}_s^{\sigma ,\tau }(\varvec{\varOmega }). $$

#### Proof

This statement follows because single-objective games are determined [26] and Algorithm 2 obtains all values by either solving single-objective instances directly (Line 3) or calling Algorithm 1, which also reduces everything to the single-objective case (Line 5 of Algorithm 1). Thus the sup-inf values $\mathbf {v}^{\mathsf {lex}}$ returned by the algorithm are in fact equal to the inf-sup values.    $\square$

By analyzing Algorithm 2, we also get the following complexity results:

#### Theorem 4

**(Complexity).** For any SG $\mathcal {G}$ and lex-objective $\varvec{\varOmega }= (\varOmega _1, \dots , \varOmega _n)$:

1. *Strategy complexity:* Deterministic strategies with $2^n - 1$ memory-classes (i.e., bit-size *n*) are sufficient and necessary for lex-optimal strategies.
2. *Computational complexity:* The lex-game decision problem ($\mathbf {v}^{\mathsf {lex}}(s_0) \ge _{\mathsf {lex}}$ $\textit{\textbf{x}}$?) is $\mathsf {PSPACE}$-hard and can be solved in $\mathsf {NEXPTIME}\cap \mathsf {coNEXPTIME}$. If *n* is a constant or $\varvec{\varOmega }$ is absorbing, then it is contained in $\mathsf {NP}\cap \mathsf {coNP}$.

#### Proof

1. For each stage, Algorithm 2 computes an MD strategy for the quantitative objective. These strategies are then concatenated whenever a new stage is entered. Equivalently, every stage has an MD strategy for every state, so as there are at most $2^n - 1$ stages (since there are *n* objectives), the strategy needs at most $2^n - 1$ states of memory; these can be represented with *n* bits. Intuitively, we save for every target set whether it has been visited. The memory lower bound already holds in non-stochastic reachability games where all *n* targets have to be visited with certainty [30].
2. The work of [41] shows that in MDPs, it is $\mathsf {PSPACE}$-hard to decide if *n* targets can be visited almost-surely. This problem trivially reduces to ours. For the $\mathsf {NP}$ upper bound, observe that there are at most $2^n - 1$ stages, i.e., a constant amount if *n* is assumed to be constant (or even just one stage if $\varvec{\varOmega }$ is absorbing). Thus we can guess an MD strategy for player $\mathsf {Max}$ in every stage. The guessed overall strategy can then be checked by analyzing the induced MDP in polynomial time [29]. The same procedure works for player $\mathsf {Min}$ and since the game is determined, we have membership in $\mathsf {coNP}$. In the same way we obtain the $\mathsf {NEXPTIME}\cap \mathsf {coNEXPTIME}$ upper bound in the general case where *n* is arbitrary.    $\square$

We leave the question whether $\mathsf {PSPACE}$ is also an upper bound open. The main obstacle towards proving $\mathsf {PSPACE}$-membership is that it is unclear if the lex-value – being dependent on the value of *exponentially* many stages in the worst-case – may actually have exponential bit-complexity.

## Experimental Evaluation

In this section, we report the results of a series of experiments made with a prototypical implementation of our algorithm.

**Case Studies.** We have considered the following case studies for our experiments:

- **Dice.** This example is shipped with PRISM-games [37] and models a simple dice game between two players. The number of throws in this game is a configurable parameter, which we instantiate with 10, 20 and 50. The game has three possible outcomes: Player $\mathsf {Max}$ wins, Player $\mathsf {Min}$ wins or draw. A natural lex-objective is thus to maximize the winning probability and then the probability of a draw.
- **Charlton.** This case study [24] is also included in PRISM-games. It models an autonomous car navigating through a road network. A natural lex-objective is to minimize the probability of an accident (possibly damaging human life) and then maximize the probability to reach the destination.
- **Hallway (HW).** This instance is based on the Hallway example standard in the AI literature [15, 38]. A robot can move north, east, south or west in a known environment, but each move only succeeds with a certain probability and otherwise rotates or moves the robot in an undesired direction. We extend the example by a target wandering around based on a mixture of probabilistic and demonic non-deterministic behavior, thereby obtaining a stochastic game modeling for instance a panicking human in a building on fire. Moreover, we assume a 0.01 probability of damaging the robot when executing certain movements; the damaged robot’s actions succeed with even smaller probability. The primary objective is to save the human and the secondary objective is to avoid damaging the robot. We use square grid-worlds of sizes $5\times 5$, $8\times 8$ and $10\times 10$.
- **Avoid the Observer (AV).** This case study is inspired by a similar example in [14]. It models a game between an intruder and an observer in a grid-world. The grid can have different sizes as in HW, and we use $10\times 10$, $15\times 15$ and $20\times 20$. The most important objective of the intruder is to avoid the observer, its secondary objective is to exit the grid. We assume that the observer can only detect the intruder within a certain distance and otherwise makes random moves. At every position, the intruder moreover has the option to stay and search to find a precious item. In our example, this occurs with probability 0.1 and is assumed to be the third objective.

**Implementation and Experimental Results.** We have implemented our algorithm within PRISM-games [37]. Since PRISM-games does not provide an *exact* algorithm to solve SGs, we used the available value iteration to implement our single-objective blackbox. Note that since this value iteration is not exact for single-objective SGs, we cannot compute the exact lex-values. Nevertheless, we can still measure the overhead introduced by our algorithm compared to a single-objective solver.

In our implementation, value iteration stops if the values do not change by more than $10^{-8}$ per iteration, which is PRISM’s default configuration. The experiments were conducted on a 2.4 GHz Quad-Core Intel^©^ Core™ i5 processor, with 4 GB of RAM available to the Java VM. The results are reported in Table 1. We only recorded the run time of the actual algorithms; the time needed to parse and build the model is excluded. All numbers are rounded to full seconds. All instances (even those with state spaces of order $10^6$) could be solved within a few minutes.

**Table 1.** Experimental Results. The two leftmost columns of the table show the type of the lex-objective, the name of the case studies, possibly with scaling parameters, and the number of states in the model. The next three columns give the verification times (excluding time to parse and build the model), rounded to full seconds. The final three columns provide the average number of actions for the original SG as well as all considered subgames $\widetilde{\mathcal {G}}$ in the main stage, and lastly the fraction of stages considered, i.e. the stages solved by the algorithm compared to the theoretically maximal possible number of stages ($2^n-1$).

Model	|*S*|	Time			Avg. actions		Stages
		Lex.	First	All	$\mathcal {G}$	$\widetilde{\mathcal {G}}$	
**R – R**							
Dice[10]	4,855	<1	<1	<1	1.42	1.41	1/3
Dice[20]	16,915	<1	<1	<1	1.45	1.45	1/3
Dice[50]	96,295	3	2	2	1.48	1.48	1/3
**S – R**							
Charlton	502	<1	<1	<1	1.56	1.07	3/3
**R – S**							
HW[$5\times 5$]	25,000	10	7.15	7	2.44	1.02	3/3
HW[$8\times 8$]	163,840	152	117	117	2.50	1.01	3/3
HW[$10\times 10$]	400,000	548	435	435	2.52	1.01	3/3
**S–R–R**							
AV[$10\times 10$]	106,524	15	<1	10	2.17	1.55, 1.36	4/7
AV[$15\times 15$]	480,464	85	<1	50	2.14	1.52, 1.36	4/7
AV[$20\times 20$]	1,436,404	281	3	172	2.13	1.51, 1.37	4/7

The case studies are grouped by the type of lex-objective, where R indicates reachability, S safety. For each combination of case study and scaling parameters, we report the state size in column |*S*|, three different model checking runtimes, the average number of actions in the original and all considered restricted games, and the fraction of stages considered, i.e. the stages solved by the algorithm compared to the theoretically maximal possible number of stages ($2^n-1$).

We compare the time of our algorithm on the lexicographic objective (Lex.) to the time for checking the first single objective (First) and the sum of checking all single objectives (All). We see that the runtimes of our algorithm and checking all single objectives are always in the same order of magnitude. This shows that our algorithm works well in practice and that the overhead is often small. Even on SGs of non-trivial size (HW[$10\times 10$] and AV[$20\times 20$]), our algorithm returns the result within a few minutes.

Regarding the average number of actions, we see that the decrease in the number of actions in the sub-games $\widetilde{\mathcal {G}}$ obtained by restricting the input game to optimal actions varies: For example, very few actions are removed in the Dice instances, in AV we have a moderate decrease and in HW a significant decrease, almost eliminating all non-determinism after the first objective. It is our intuition that the less actions are removed, the higher is the overhead compared to the individual single-objective solutions. Consider the AV and HW examples: While for AV[$20\times 20$], computing the lexicographic solution takes 1.7 times as long as all the single-objective solutions, it took only about 25% longer for HW[$10\times 10$]; this could be because in HW, after the first objective only little nondeterminism remains, while in AV also for the second and third objectives lots of choices have to be considered. Note that the first objective sometimes (HW), but not always (AV) needs the majority of the runtime.

We also see that the algorithm does not have to explore all possible stages. For example, for Dice we always just need a single stage, because the SG is absorbing. For charlton and HW all stages are relevant for the lex-objective, while for AV 4 of 7 need to be considered.

## Conclusion and Future Work

In this work we considered simple stochastic games with lexicographic reachability and safety objectives. Simple stochastic games are a standard model in reactive synthesis of stochastic systems, and lexicographic objectives let one consider multiple objectives with an order of preference. We focused on the most basic objectives: safety and reachability. While simple stochastic games with lexicographic objectives have not been studied before, we have presented (a) determinacy; (b) strategy complexity; (c) computational complexity; and (d) algorithms; for these games. Moreover, we showed how these games can model many different case studies and we present experimental results for them.

There are several directions for future work. First, for the general case closing the complexity gap ($\mathsf {NEXPTIME}\cap \mathsf {coNEXPTIME}$ upper bound and $\mathsf {PSPACE}$ lower bound) is an open question. Second, the study of lexicographic simple stochastic games with more general objectives, e.g., quantitative or parity objectives poses interesting questions. In particular, in the case of parity objectives, there are some indications that the problem is significantly harder: Consider the case of a reachability-safety lex-objective. If the lex-value is (1, 1) then both objectives can be guaranteed almost surely. Since almost-sure safety is sure safety, our results imply that sure safety and almost-sure reachability can be achieved with constant memory. In contrast, for parity objectives the combination of sure and almost-sure requires infinite-memory (e.g, see [21, Appendix A.1]).
